# Disparities in Dental Service Use Among Adult Populations in the United States

**DOI:** 10.1093/geroni/igab046.794

**Published:** 2021-12-17

**Authors:** Yan Yan Wu, Wei Zhang, Bei Wu

**Affiliations:** 1 University of Hawaii at Manoa, University of Hawaii at Manoa, Hawaii, United States; 2 University of Hawaii at manoa, Honolulu, Hawaii, United States; 3 New York University, New York, New York, United States

## Abstract

This paper aimed to examine disparities of dental service utilization for younger (20-49), middle-aged (50-64), and older adults (65+), among Whites, Hispanics, Blacks, Asians, American Indians or Alaska Natives (AIAN), and Native Hawaiian or other Pacific Islanders (NHOPI). Weighted logistic regression models were conducted to analyze nine waves of data (2002-2018) from the Behavioral Risk Factor Surveillance System. Results show that the all-wave average prevalence was 71% and racial/ethnic disparities increased with age. Black older adults had the lowest level of dental service utilization (65%), comparing to the two highest groups: White older adults (79%) and Asian older adults (76%). The younger adult populations had low prevalences with the lowest among Asians (65%). The AIAN and NHOPI all age groups tended to have average or below average prevalences. Health policy, federal funding, and community-based programs should address needs of dental service utilization for racial/ethnic minorities including Blacks, AIANs, and NHOPIs.

